# Relation of intelligence quotient and body mass index in preschool children: a community-based cross-sectional study

**DOI:** 10.1038/nutd.2015.27

**Published:** 2015-08-10

**Authors:** A A Tabriz, M-R Sohrabi, S Parsay, A Abadi, N Kiapour, M Aliyari, F Ahmadi, A Roodaki

**Affiliations:** 1 Department of Health Policy and Management, The Gillings School of Global Public Health, University of North Carolina at Chapel Hill, Chapel Hill, NC, USA; 2 Social Determinants of Health Research Center and Department of Community Medicine, School of Medicine, Shahid Beheshti University of Medical Sciences, Tehran, Iran; 3 Community Medicine Department, Shahid Beheshti University of Medical Sciences, Tehran, Iran; 4 Department of Microbiology and Immunology, UNC School of Medicine, University of North Carolina at Chapel Hill, Chapel Hill, NC, USA; 5 Department of Radiology, The Johns Hopkins University School of Medicine, Baltimore, MD, USA; 6 Supélec, Gif-sur-Yvette, Paris, France

## Abstract

**Objective:**

Overweight and obesity in children is a global problem. Besides physical effects, obesity has harmful psychological effects on children.

**Methods:**

We carried out cross-sectional community-based study to investigate the relation between body mass index (BMI) and cognitive functioning in preschool children. Thirteen socioeconomical elements of 1151 children were measured and analyzed based on their intelligence quotient (IQ) test results. Thirteen out of 33 provinces were selected randomly, and schools were selected as clusters in rural and urban areas. Descriptive statistics, *t*-test, analysis of variance and regression were used when appropriate.

**Results:**

Our analysis showed that IQ was associated with household income, place of residence, delivery type, type of infant feeding and father's and mother's education level (*P*<0.001 for all). Using penalized linear regression for eliminating the impact of confounding factor, our study shows that, living in metropolitan (*β*=2.411) and urban areas (*β*=2.761), the level of participants' father's education (*β*=5.251) was positively and BMI (*β*=−0.594) was negatively related with IQ test results.

**Conclusions:**

The findings of the present study showed that a lower IQ score is associated with higher BMI. However, this relation appears to be largely mediated when the socioeconomic status was considered.

## Introduction

Prevalence of overweight and obesity among children has been noticeably rising over the past two decades.^1^ Overweight and obesity in children have become a global problem^2, 3^ and no longer limited to high-income societies,^4^ but prevailing among developing countries, such as the Middle East, as well.^5^ Data on young people living in the Middle East are limited in this regard;^6^ however, it is widely assumed that the picture of health and nutritional status in this region has changed during the past four decades.^7^ The cardiometabolic consequences of obesity, especially hypertension,^8^ impaired glucose metabolism,^9^ extra stress on weight-bearing joints,^10^ liver disease^11^ and asthma, have been a cause of special concern.^12^

Besides the physical effects, being overweight may have harmful psychological effects on children, such as lowering self-esteem, affecting relationships with peers and social problems.^13, 14^ Despite it is well established that both genetic and environmental factors have influence on cognitive abilities,^15, 16^ the relation between childhood body mass index (BMI) status and cognitive functioning in a population of normal children has been the subject of limited research.^17, 18^ Although the available evidence suggests an inverse association between childhood intelligence quotient (IQ) and adulthood obesity,^19^ the effect of obesity on cognitive abilities in children has remained under study.

We carried out cross-sectional community-based study to investigate the relation between BMI and IQ test results in preschool children.

## Materials and methods

This cross-sectional community-based study was performed at the national level among preschool children aged 6–7 years old, between 25 April 2009 and 19 May 2013.

The project team selected samples from preschool children by multistage random cluster sampling from the preliminary schools of 13 out of 33 provinces of Iran. Participants were chosen from Tehran (metropolitan area, 605.3 km^2^; a population of 11 000 000), Karaj (urban area, 141.8 km^2^; a population of 971 150), Mashhad (urban area, 458 km^2^; a population of 2 907 316), Tabriz (urban area, 342 km^2^; a population of 1 398 060), Mehriz (suburban area; a population of 74 000), Dezful (suburban area; a population of 235  819), Kermanshah (urban area; a population of 965 665), Zabol (suburban area; a population of 128 476), Bashagard (rural area with 31 subrural area; a population of 31 502), Horand (rural area with 13 subrural area; a population of 29 384), Deyhok (rural area with 17 subrural area; a population of 27 305), Delphan (rural area with 7 subrural area; a population of 19 000) and Zarneh (rural area with 6 subrural area; a population of 6300). We selected these 13 clusters to cover the geographical and cultural differences in the Iranian population and to guarantee the generalizability of the results. From each area, proportional to the population size, 1–10 schools (totally 74 schools) were selected randomly. In the next stage, in each randomly selected school, a probability proportional to the population size in two-stage cluster sample of students was selected. Then, the students within the schools were coded and randomly selected using random number table. The initial sample for this study consisted of 1352 children, from which 201 ones were excluded.

Exclusion criteria were mental retardation (*n*=0), any chronic medical problem such as asthma, pediatric type 1 diabetes mellitus and anemia (*n*=18), long-term medication use for any health disorder (*n*=17), signs compatible with genetic syndromes such as abnormal face (*n*=1), history of any kind of infectious or non-infectious hepatic disorder (*n*=2), history of familial hyperlipidemia (*n*=1), endocrine disorders (*n*=0) any physical disability (*n*=5), obesity secondary to genetic disorders (*n*=1), hypothyroidism (*n*=0), living with single parents (*n*=28), previous history of autism (*n*=1), a neurological disorder or hearing loss (*n*=1) and any developmental neurologic or psychiatric alterations (*n*=1). Children under special diets (*n*=12), children who have been examined by means of the Wechsler Intelligence Scale for Children test in the past 6 months (*n*=11), identical twins (*n*=12), 24 children because of the lack of data and 78 children because of their or their parents refusal to participate in the study were also excluded.

At first, detailed oral information was presented to children and parents and written informed consent was obtained from the parents of all eligible study participants. Later, under the supervision of expert health-care professionals, each child and one of the parents were invited to the school, and the self-administered questionnaire were filled at the same time. Age (date of birth based on child's ID card), gender, history of any previous education for the child (by family member or kindergarten), household size and area of residence (metropolitan, urban, suburban, rural, subrural), age and educational level of parents (education was defined as the total number of years of education, leading us to three categories: 0–12, low educated; 12–16, moderate educated; and >16 years of education, highly educated), type of childbirth (cesarean section or natural vaginal delivery), type of infant feeding (breastfeeding or formula) and minimum household income (to obtain estimates of household income, we mapped each subject's address to a census tract; we used the Iran Census data from 2011 to assign the median household income for that census tract to the individual subject) were documented at study entry. The data entry staff entered data for all forms and questionnaires two times and rechecked for completeness and inconsistencies. After filling a coded questionnaire by parents, height and weight of each child were measured. Height was measured while the subject was standing without shoes and socks to the nearest 0.1 cm, using a specially designed portable stadiometer with a spirit level to ensure that it was parallel to the hard flat floor during measurement. Weight was measured while the child was standing and wearing light clothes to the nearest 0.1 kg, using a calibrated digital scale, which was recalibrated between each 25 measurements. For the tests, we were assisted by 26 pediatric nurses who were trained for 10 h by a physician with 12 years of experience in the field. Additionally, nurses were given 1-h-long test before the beginning of the session to ensure their testing skills. Two trained nurses under the supervision of a physician took all measurements and one of the research team members supervised the tests in every school at each site. Two more physicians reviewed all the raw data two times.

BMI was computed as weight in kilograms divided by the square of height in meters. The BMI cutoff points used were those from the Centers for Disease Control and Prevention (CDC).^20^ To standardize BMI levels, conversion to a BMI *Z*-score was performed based on the CDC growth charts. Although significantly overweight is often characterized as obese, the CDC criteria and categorical labels were adopted as follows: (1) underweight, BMI for age at <5th percentile; (2) normal weight, BMI for age at the 5th percentile to <85th percentile; (3) overweight risk, BMI for age at the 85th percentile to <95th percentile; and (4) overweight, BMI for age at 95th percentile.

After that, we applied the Wechsler Intelligence Scale for Children-Fourth Edition, Full-Scale IQ^21^ to assess the general thinking and reasoning skills of children. Full-scale IQ scores were classified in the following seven levels: >130, very superior; 120–129, superior; 110–119, normal-brilliant; 90–109, normal; 80–89, normal-awkward; 70–79, borderline; and <69, mental retardation. A pediatric psychologist who was unaware of the aim of the research measured the IQ level in the participating children. A time is then set to administer the test, which takes on an average 60–90 min duration.

Medical Research Ethics Committee of Shahid Beheshti University of Medical Science granted approval for the study. The Data and Safety Monitoring Board of the project closely supervised the quality control and quality assurance of the survey at the national level and there was no interventional component to this study.

The entire coded questionnaire is matched with the same coded IQ test result. Data are presented as percentages, percentiles, means and s.d. Testing of continuous variables was made by *t*-test for two comparisons or by one-way analysis of variance for multiple comparisons. In this case, significance between sample means was measured by Tukey's HSD (honest significant difference) *post hoc* test. The strength of association between variables was calculated by coefficients from multivariate linear regression models. In all cases, the significance level was *α*=0.050. All statistical analyses were performed using the SPSS software 16 (SPSS Inc., Chicago, IL, USA).

## Result

A total of 1151 children participated in our study, of which 597 (51.9%) were boys and 554 (48.1%) were girls. Birth rank of 187 (16.2%) children was first, 302 (26.2%) was second, 347 (30.1%) was third, 180 (15.6%) was fourth, 116 (10.1%) was fifth and 19 (1.6%) was sixth and above. The mean size of family was 5.73±1.54, with a range of 3–12 members. The mean age of fathers of participants was 40.40±6.93 years and the mean age of mothers of participants was 35.59 ± 5.73 years. Table 1 shows the basic characteristics of the participants.

The mean of IQ was 99.46±2.12 with the range 71–131. Our analysis showed that IQ was strongly associated with household income, place of residence, previous education, delivery type, infant feeding and parent's educational degree as presented in Table 2.

The mean of IQ based on place of residence (in urban and suburban areas (*P*=0.474) and rural and subrural areas (*P*=0.990)) was not significantly different. Therefore, for the next analysis we merged urban and suburban areas together as rural and subrural areas. For eliminating the impact of confounding factors on IQ, we used the forward selection linear regression. The technique we used is a penalized linear regression called 'Lasso',^22^ and to solve that we used the coordinate descent method. Our analysis stopped at the fourth step, living in metropolitan and urban areas, as the level of participants' father's education was positively associated with IQ. When BMI was considered, an inverse association was observed between IQ score and BMI as presented in Table 3.

**Figure 1:**
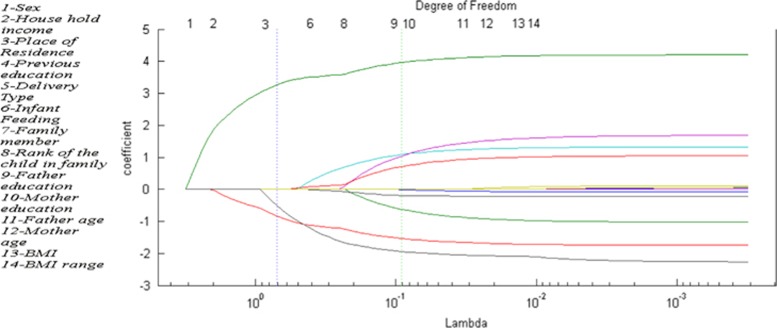
Regularization path of Lasso coefficient.

The weight of the penalty term in Lasso depends on a regularization coefficient (*λ*).^22^ Figure 1 shows the regularization path, which shows the variations of the explanatory variable coefficients versus *λ*. To obtain that figure, we solved the Lasso for several values of *λ*. Each line shows variation coefficient versus *λ*. It can be seen that for high values of *λ*, no variable is included in the model, whereas for small values of *λ*, all variables were included. To select the final solution, we need to choose *λ* in a mathematically useful way. As Figure 1 shows, there are two vertical lines. The green line indicates the solution with the minimum mean-squared error and the blue vertical line indicates the value of *λ* with the minimum mean-squared error of cross-validation, where the obtained solution is sparse.

The non-zero coefficients are place of residence=0.9, father's educational level=3.33 and BMI range=−0.65. The reason why, for example, father's educational level was considered and mother's educational level was not can be found in the correlation matrix of the variables, which shows that father's and mother's educational level was strongly correlated (0.82).

## Discussion

Findings from a succession of studies indicated that childhood intelligence is inversely associated with several health outcomes in later life.^23, 24, 25, 26, 27^ Owing to similar association between childhood obesity and dire health consequences in adulthood,^28, 29, 30, 31, 32^ the main aim of this study was to expand our understanding of the relation between children's physical status and cognitive ability by considering the impact of confounding socioeconomic factors. By adjusting the impact of these factors, the findings of the present study showed that a lower IQ score is associated with abnormal BMI, especially higher BMI. However, this relation appears to be largely mediated when the socioeconomic status was considered.

Our findings showed that the IQ score of children was in a high positive relation with maternal and paternal education, which is comparable with other studies.^33, 34^ One implication could be the effect of genetic factors on children's IQ.^15, 16, 35, 36^ Other implications could be better mother's nutrition during pregnancy, the importance of children's education among highly educated families and higher rate of preschool education among families with higher educational level.

Our study showed that children born by cesarean section have higher IQ compared with naturally born children, which is somehow different from the results reported from the previous studies.^37^ This might be because of higher rate of cesarean section in women who are more educated and urban citizens and not just because of the type of delivery.^38^

Consistent with many prior studies,^39, 40, 41^ our study indicated that children who feed on their mothers' milk during their infancy have higher IQ. This might be because breastfeeding was more common among women of higher socioeconomic groups or because of direct effect of human milk on children's brain development.^42^

Our study revealed that children who lived in better socioeconomic status such as wealthier and smaller families or children who live in bigger cities have higher IQ, which is in line with previous studies, which discussed about the impact of socioeconomic status on IQ in young children.^16, 43, 44, 45, 46, 47, 48, 49^ Our linear regression showed that the area of residence has a significant relation with IQ (Table 3). Exposure to the more complicated environment, benefiting from parents who are more educated, lesser exposure to stressful life events and a lesser impact of these events and lower cortisol secretion^50^ could be the underlying causes.

As might be expected,^51, 52, 53, 54^ no difference in IQ score was observed based on gender, ethnic and parents age.

With regard to the complex impact of socioeconomic factors on both children's BMI and IQ,^55, 56, 57, 58^ it is likely that no single socioeconomic factor can fully explain the relation between children with BMI and their IQ. Some studies^59, 60, 61^ identified a strong evidence for an inverse association between child overweight and academic performance on mathematics, reading/language arts, science and social studies by standardized tests across diverse samples of children and adolescents. Although other studies doubted on this relation by considering the influence of socioeconomic status,^62, 63^ our study showed a significant relation between IQ scores of children and their BMI. IQ–obesity association remained statistically significant after adjusting for the type of delivery, type of infancy feeding, area of residence, household income and parents education, although it was attenuated considering these factors.

Last but yet important, the findings of the present study indicate an overall prevalence rate of 5.7% for obesity, 16.2% for overweight and 14.7% for underweight in Iranian population of children. The results of this study showed a significant rise in the prevalence of weight disorders among Iranian children during the previous decade.^6, 7, 64, 65, 66^ Although the high obesity prevalence among Iranian adults^67, 68^ and dreadful consequences of obesity on adult's cognitive functions^69, 70, 71, 72^ was well established, new results alarmed the Iranian policy makers that they are now constrained to instantaneously tackle the burden of over- and undernutrition problems in Iranian population of children. The problem of childhood obesity is too extensive and the consequences too severe and costly to postpone intervention.^73^ These findings also highlight the importance of assessing and providing service to children who are underweight, which, especially, has higher prevalence in rural areas. The double burden of nutritional disorders among young children warrants a multifaceted national policy health-care system.^1, 2, 3, 4, 5, 6, 7, 74, 75^ Owing to the rapid lifestyle change in Iran and the still existing belief among families that childhood overweight is a sign of health,^7^ and by considering the success of preventive programs,^76, 77, 78, 79^ it is time for Iranian policy makers to overlook Iran's national strategies and focus their efforts on implementing more preventive programs on preschool aged children.

As described, the strengths of this study include in its size, consisting of both urban and rural populations, sound representativeness of the national population, the sex-specific analyses, rigorous methodology and measurements that guarantee the accuracy of data and the wide range of covariance data. There are a few limitations to this study. First, we adjusted for various factors related to growth and cognition; however, there were additional variables that we could not consider, such as diet, physical activity levels, paternal IQ and measures of attachment and parenting skills. Each of these is known to be important in predicting growth and cognitive development.^38^

## Figures and Tables

**Table 1:** Baseline characteristics of participants based on their gender

*Characteristic*	*Boy (%)*	*Girl (%)*	*Total (%)*	P*-value*
*Household income*				
Low	223 (37.4)	201 (36.3)	424 (36.8)	0.691
Moderate	235 (39.4)	220 (39.7)	455 (39.5)	
High	139 (23.2)	133 (24.0)	272 (23.6)	

*Area of residence*				
Metropolitan	102 (17.1)	87 (15.7)	189 (16.4)	0.385
Urban	173 (29.0)	156 (28.2)	329 (28.6)	
Suburban	142 (23.7)	132 (23.8)	274 (23.8)	
Rural	130 (21.8)	130 (23.5)	260 (22.6)	
Subrural	50 (8.4)	49 (8.8)	99 (8.6)	

*Delivery type*				
NVD	278 (46.6)	275 (49.6)	553 (48)	0.298
C/S	319 (53.4)	279 (50.4)	598 (52)	

*Infant feeding*				
Breast feeding	342 (57.3)	305 (55.0)	647 (56.2)	0.446
Formula	255 (42.7)	249 (45.0)	504 (43.8)	

*Father's education*				
Low	225 (37.7)	209 (37.7)	434 (37.1)	0.724
Moderate	326 (54.6)	309 (55.8)	635 (55.2)	
High	46 (7.7)	36 (6.5)	82 (7.1)	

*Mother's education*				
Low	255 (42.7)	219 (39.5)	474 (41.2)	0.311
Moderate	329 (55.1)	323 (58.3)	652 (56.6)	
High	13 (2.2)	12 (2.2)	25 (2.2)	

*History of previous education*				
Positive	497 (83.2)	496 (89.5)	993 (86.3)	0.205
Negative	100 (16.8)	58 (10.5)	158 (13.7)	

*BMI range*				
Under weight	90 (15.1)	79 (14.3)	169 (14.7)	0.935
Normal	379 (63.5)	351 (63.4)	730 (63.4)	
Overweight	93 (15.6)	93 (16.8)	186 (16.2)	
Obese	35 (5.9)	31 (5.6)	66 (5.7)	

Abbreviations: BMI, body mass index; C/S, cesarean section; NVD, normal vaginal delivery.

**Table 2:** Comparison of the mean of IQ in different variables

*Variable*	*IQ*	P*-value*	t *(or F)-value*
*Sex*		0.927	−0.092
Boys	99.44±9.38		
Girls	99.49±9.85		
			
*Household income*		<0.001	22.468
Low	97.25±9.42		
Moderate	100.00±9.37		
High	102.03±9.61		
			
*Place of residence*		<0.001	24.988
Metropolitan	103.50±10.34		
Urban	100.92±8.96		
Suburban	99.68±9.29		
Rural	95.98±9.29		
Subrural	95.47±7.32		
			
*Previous education*		<0.001	6.577
Positive	100.19±9.59		
Negative	94.89±8.37		
			
*Delivery type*		<0.001	6.745
C/S	101.27 ±9.56		
NVD	97.57 ±9.28		
			
*Infant feeding*		<0.001	3.796
Breast feeding	100.67 ±10.01		
Formula	98.52±9.17		
			
*Father's education*		<0.001	87.096
Low	96.65±9.03		
Moderate	99.93±8.57		
High	110.73±11.31		
			
*Mother's education*		<0.001	65.540
Low	96.71±9.05		
Moderate	100.88±9.08		
High	114.92±10.76		

Abbbreviations: C/S, cesarean section; IQ, intelligent quotient; NVD, normal vaginal delivery.

**Table 3:** Linear regression models

*Models*	*Independent variables*	β	*S.e.*β	t*-Value*	P*-value*	R^*2*^
Model 1	Constant	90.568	0.807	112.161	<0.001	0.106
	Education father	5.251	0.450	11.679	<0.001	
						
Model 2	Constant	90.970	0.814	111.730	<0.001	0.114
	Education father	4.78	0.472	10.132	<0.001	
	Place (metropolitan)	2.411	0.759	3.179	0.002	
						
Model 3	Constant	97.326	2.083	48.390	<0.001	0.123
	Education father	5.132	0.480	10.680	<0.001	
	Place (metropolitan)	2.718	0.760	3.576	<0.001	
	BMI	−0.594	0.134	−3.453	0.001	
						
Model 4	Constant	99.519	2.083	47.784	<0.001	0.134
	Education father	4.119	0.548	7.517	<0.001	
	Place (metropolitan)	5.121	0.989	5.180	<0.001	
	BMI	−0.601	0.136	−4.422	<0.001	
	Place (urban and suburban)	2.761	0.732	3.771	<0.001	

Abbreviation: BMI, body mass index.
